# Characteristics of older adults using patient web portals to view their DXA results

**DOI:** 10.1186/s12911-019-0904-y

**Published:** 2019-09-18

**Authors:** Stephanie Edmonds, Yiyue Lou, Brandi Robinson, Peter Cram, Douglas W. Roblin, Nicole C. Wright, Kenneth Saag, Fredric D. Wolinsky

**Affiliations:** 1 Carver College of Medicine, Department of Internal Medicine, University of Iowa, 200 Newton Rd. 5231 WL, Iowa City, IA 52242 USA; 2 CADRE, Iowa City VA Health System, Iowa City, IA USA; 3 College of Public Health, Department of Biostatistics, University of Iowa, Iowa City, IA USA; 4 Kaiser Permanente, Center for Clinical and Outcomes Research, Atlanta, GA USA; 5 College of Medicine, Department of Internal Medicine, University of Toronto, Toronto, Canada; 6 University of Toronto Health Network, Toronto, Canada; 7 Mid-Atlantic Permanente Research Institute, Rockville, MD USA; 8 School of Public Health, Department of Epidemiology, University of Alabama at Birmingham, Birmingham, AL UK; 9 School of Medicine, Department of Rheumatology, University of Alabama at Birmingham, Birmingham, AL UK; 10 College of Public Health, Department of Health Management and Policy, University of Iowa, Iowa City, IA USA; 11 College of Nursing, University of Iowa, Iowa City, IA USA

## Abstract

**Background:**

Sharing test results with patients via patient web portals is a new trend in healthcare. No research has been done examining patient web portal use with bone density test results. The objective of our study was to identify patient characteristics associated with the use of patient web portals to view their bone density test results.

**Methods:**

A secondary analysis of data from a pragmatic randomized controlled trial of 7749 participants ≥50 years old that had presented for a dual energy X-ray absorptiometry (DXA) bone density test. Patients were interviewed at enrollment and 12 weeks later. Multivariable logistic regression identified patient characteristics that differentiated those who used the web portal from those who did not.

**Results:**

Our sample included 4669 patients at the two (University of Iowa [UI], and Kaiser Permanente of Georgia [KPGA]) clinical sites that had patient web portals. Of these patients, 3399 (72.8%) reported knowing their test results 12 weeks post-DXA, with 649 (13.9%) reporting that they viewed their DXA results using the web portal. Web portal users were more likely to be from UI than KPGA, and were younger, more educated, had higher health literacy, had osteopenia, and had the same sex as their referring physician (all *p* < 0.05).

**Conclusion:**

Only 19.1% of the 3399 patients who knew their DXA results used the available patient web portals to find out about them. Web portal users differed from non-users on several characteristics. This suggests that simply making patient web portals available for use may not be sufficient to appreciably enhance patient awareness of their test results. Based on these findings, a better understanding of the reasons why older, less educated, and less activated patients do not access their test results through patient web portals is needed.

## Introduction

In 2009, the Centers for Medicare and Medicaid Services (CMS) established the Electronic Health Records Incentive Programs “to promote the adoption and meaningful use of interoperable health information technology (HIT) and qualified electronic health records (EHRs)” [1]. One HIT approach for achieving this goal is sharing information in the EHR with patients via web portals. The advantages of providing patients with access to their test results are numerous, including fostering self-efficacy and patient activation as well as preventing test results from falling-through-the cracks, which is a common medical error and a threat to patient safety [2]. For this reason, the Joint Commission established goals for communicating test results [3]. Patient portals, like *MyChart* from *Epic,* is one method patients can access their test results. Patient web portals have been shown to improve patient satisfaction and health care quality [4–10].

A commonly performed test among older adults is dual energy x-ray absorptiometry (DXA), which is the standard screening tool for identifying patients at increased risk for hip or other fragility fractures. But even when a diagnosis of osteoporosis is made from the DXA, patients do not always receive appropriate pharmacotherapy consistent with treatment guidelines [11]. Indeed, one study found that two-thirds of patients with DXA results indicating osteoporosis did not receive appropriate pharmacotherapy. And using chart review methods, another study revealed among 23% of abnormal DXA results there was no evidence that the patient’s clinician had reviewed their DXA results or communicated them to their patients [11]. One way to increase patient access to their DXA results would be to use web portals to communicate test results directly to patients. To our knowledge, however, there have not been any studies of patients’ use of web portals for learning about their DXA results. Sharing DXA results with patients via web portals may also activate them to follow-up with their health care provider, especially if their results are abnormal.

Web portals are relatively new in medicine. A recent systematic review found low use rates for patient web portals across a number of studies [12]. The main reasons given included concerns about confidentiality, poor awareness of web portals, and inadequate user friendliness. Overall, patients who did use web portals as part of the health care were typically younger, and more likely to be White, affluent, women with chronic disease [12].

Our objective was to examine patient and clinical factors associated with the use of web portals to review their DXA results. We did this using data from the Patient Activation after DXA Result Notification clinical trial (PAADRN- https://clinicaltrials.gov/ct2/show/NCT01507662), Identifying the characteristics of web portal users, specifically for DXA results, may provide the information needed to inform practitioners about the appropriate ways to communicate DXA results, as well as provide insight into how best to craft interventions to increase web portal use.

## Methods

### PAADRN study

This manuscript describes a secondary data analysis of data from the PAADRN study, a pragmatic, randomized controlled trial conducted at the DXA clinics of three medical centers in the United States (U.S.)—the University of Iowa (UI), the University of Alabama at Birmingham (UAB), and Kaiser Permanente of Georgia (KPGA) [13]. PAADRN’s main purpose was to examine the effects a tailored DXA result notification letter accompanied by a bone-health educational brochure mailed to patients on guideline-concordant osteoporosis pharmacotherapy., Eligible patients were those presenting for DXA who were 50 years old or older and could read and speak English. We excluded patients who had significant cognitive impairments which would prevent them from providing informed consent, prisoners, and those with visual or hearing impairments. Institutional Review Boards from all three sites approved the study and we collected written or verbal informed consent per institutional policy.

### Survey instrument

As previously described [13], study-specific RAs asked participants questions about their demographics, bone and general health histories, health literacy, subjective numeracy (α = 0.811) [14], preference for self-care (α = 0.771) [15], patient activation (PAM, α = 0.661) [16], and measures of their osteoporosis-related knowledge (α = 0.685) [17, 18], health beliefs (α = 0.737) [19], and self-efficacy (α = 0.958) [20]. About 12-weeks later, trained interviewers from the Iowa Social Science Research Center re-contacted patients by telephone and asked them a series of questions related to their DXA. In particular, patients were asked if they knew their DXA results, and if they did, how it was that they came to know about their DXA results. Patients could select more than one response option. The response options for this latter question were:

- meeting with their provider in person
- talking to their provider over the phone
- exchanging emails with their provider
- receiving a mailed letter with their results (a letter was mailed to all intervention participants)
- seeing their results on an electronic medical record or portal
- getting their results from the DXA technician
- or by some other means.

Once participants identified the methods by which they received their DXA results, the interviewers then asked them to indicate the order in which they received the results (e.g. which method was the first, second, third, etc., by which you learned of your results). A more detailed description of the interview protocol is available elsewhere [13].

### Analytic sample

We limited the current analysis to PAADRN participants from UI and KPGA because both sites had web portals available at the time of PAADRN enrollment. UAB did not have a web portal for the entire study period, and thus UAB patients were excluded. Web portal assignment was not randomized in this study. Both UI (since June 2010) and KPGA (since 2005) offered patients access to *Epic’s* (Verona, WI) web portal (known as *MyHealthManager* for KPGA and *MyChart* for UI). Of the 3185 UI patients who were potentially eligible for PAADRN, administrative records indicated that 62.6% had activated their *MyChart* accounts. Similarly, of 15,642 potentially eligible patients at KPGA, administrative records indicated that 65% had activated their *MyHealthManager* accounts. We further limited the analytic sample to PAADRN participants who reported knowing what their test results were at the time of their 12-week survey.

### Statistical analysis

We first used bivariable analysis to compare all web portal users with all non-web portal users. This included a comparison of demographic characteristics (clinical site, age, sex, race: white vs. not white, and education), patient health beliefs (health literacy, numeracy, patient activation, preference for self-care [14]), health characteristics (self-reported history of low bone density or osteoporosis, self-rated health, and a number of additional chronic conditions), DXA ordering provider characteristics (intervention assignment, provider’s gender, and whether the provider was the same sex as the patient). Continuous variables (age, health literacy, numeracy, patient activation scores, and self-care preferences) were categorized and some categorical variables (race, education, number of chronic conditions) were collapsed to improve interpretation.

Next, among web portal users we distinguished between three groups. The first group of web portal users included those who reported only learning about their results from the web portal. The second group included those who used the web portal as their first method to learn of their results, but also learned about their results later on from another source. The third group included those who first learned about their results from another method, but also learned about their results subsequently from the web portal. We compared these three groups on the demographic characteristics, patient health beliefs, health, and provider characteristics to determine whether pooling these groups was appropriate.

Finally, we used multivariable logistic regression with any web portal use as the outcome, and all of the predictors that were used in the bivariable analyses. Adjusted odds ratios (AORs) and 95% CIs were calculated for each predictor. Case-wise deletion was used to address missing data. To examine the heterogeneity of treatment effects (HTEs) involving study site, we conducted sensitivity analyses by including interactions of site vs. all of the other predictors one at a time in the adjusted models. All analyses were performed using SAS 9.4, Cary, NC.

## Results

### Study population

PAADRN’s CONSORT flow chart and descriptive baseline data for all 7749 participants can be found elsewhere [21]. Of the 4669 patients enrolled at UI and KPGA, 4005 (85.8%) completed the 12-week interview, and 84.9% of those (*N* = 3399) knew the results of their DXA (Table 1). Overall, 13.9% (*n* = 649) of participants used the web portal to view their DXA results.

**Table 1:** Characteristics of those viewing DXA results via web portal vs. those not viewing DXA results via web portal among patients reported knowing their DXA results (*N* = 3399)

Characteristic	Viewed DXA results via web portal, N (%) = 649 (19.1%)	Did not view DXA results via web portal N (%) =2750 (80.9%)	*P*-value
Demographics			
Site			
UI	385 (29.26%)	931 (70.74%)	< 0.001
KPGA	264 (12.67%)	1819 (87.33%)	
Age			
Mean (SD)	64.4 (7.4)	66.5 (8.1)	< 0.001
< 65	313 (22.66%)	1068 (77.34%)	< 0.001
65–74	270 (18.38%)	1199 (81.62%)	
> 75	66 (12.02%)	483 (87.98%)	
Gender of patient			
Male	113 (17.15%)	546 (82.85%)	0.157
Female	536 (19.56%)	2204 (80.44%)	
Race			
White	570 (21.19%)	2120 (78.81%)	< 0.001
Non-white	79 (11.14%)	630 (88.86%)	
Education			
High school or less	88 (10.92%)	718 (89.08%)	< 0.001
Some college	197 (18.02%)	896 (81.98%)	
Completed college	157 (21.3%)	580 (78.7%)	
Graduate school	204 (27.79%)	530 (72.21%)	
Patient health personal characteristics			
Health Literacy			
Low	54 (12.08%)	393 (87.92%)	< 0.001
High	592 (20.27%)	2328 (79.73%)	
Subjective Numeracy			
Low	160 (13.86%)	994 (86.14%)	< 0.001
Medium	213 (18.85%)	917 (81.15%)	
High	273 (25.11%)	814 (74.89%)	
Patient Activation Score			
Level 1 (0–42.5)	50 (15.2%)	279 (84.8%)	0.003
Level 2 (47.4–52.9)	100 (16.05%)	523 (83.95%)	
Level 3 (56.4–66.0)	302 (19.28%)	1264 (80.72%)	
Level 4 (68.5–100)	194 (22.61%)	664 (77.39%)	
Krantz Information Scale			
Low	164 (14.84%)	941 (85.16%)	< 0.001
Medium	236 (20.36%)	923 (79.64%)	
High	246 (22.18%)	863 (77.82%)	
Krantz Behavioral Scale			
Low	187 (15.7%)	1004 (84.3%)	< 0.001
Medium	193 (18.16%)	870 (81.84%)	
High	261 (23.66%)	842 (76.34%)	
Participant health characteristics			
History of Osteoporosis			
Normal	320 (16.04%)	1675 (83.96%)	< 0.001
Osteopenia	206 (26.68%)	566 (73.32%)	
Osteoporosis	123 (19.46%)	509 (80.54%)	
Self-rated Health			
Poor	17 (17.17%)	82 (82.83%)	0.007
Fair	68 (15.96%)	358 (84.04%)	
Good	215 (17.68%)	1001 (82.32%)	
Very good	245 (19.84%)	990 (80.16%)	
Excellent	104 (24.94%)	313 (75.06%)	
Number of Chronic Conditions			
None	305 (19.28%)	1277 (80.72%)	0.485
1–2	313 (19.31%)	1308 (80.69%)	
> 2	31 (15.82%)	165 (84.18%)	
Provider characteristics			
Intervention			
Intervention	316 (17.86%)	1453 (82.14%)	0.057
Usual care	333 (20.43%)	1297 (79.57%)	
Patient Provider’s Gender			
Male	208 (16.72%)	1036 (83.28%)	0.007
Female	441 (20.46%)	1714 (79.54%)	
Gender of patient and provider			
Female providers and female patients	398 (21.37%)	1464 (78.63%)	0.001
Female providers and male patients	43 (14.68%)	250 (85.32%)	
Male providers and male patients	70 (19.13%)	296 (80.87%)	
Male providers and female patients	138 (15.72%)	740 (84.28%)	

### Unadjusted associations with web portal use

Participants who knew their DXA results and reported using the web portal as one method of viewing their results were more likely to be from UI, younger, White, more highly educated, have higher health literacy and numeracy, have higher patient activation scores, have higher health information-seeking preferences. Additionally, web portal users differed on health-related characteristics such as those with history of osteopenia or osteoporosis, better self-rated health, and a female provider or a provider of the same gender than participants who did not use the web portal to view their DXA results (Table 1).

### Method of learning of DXA result

Of the 649 participants who used the web portal, 192 (29.6%) learned of their DXA results only through the web portal, 230 (35.4%) learned of their DXA results by another method as well but through the web portal first, and 227 (35.0%) learned of their DXA results from another method first but viewed the result later via the web portal (Table 2). The three groups did not differ statistically on any characteristic; thus, it was appropriate for us to combine all three groups into one ‘web portal use’ group.

**Table 2:** Characteristics of web portal users who first learned their DXA results through different sources (*N* = 649)

Characteristic	Web portal only, N (%) = 192 (29.6%)	Web portal first, N (%) = 230 (35.4%)	Web portal, but not first method reported, N (%) = 227 (35.0%)	*P*-value
Demographics				
Site				
UI	117 (30.39%)	132 (34.29%)	136 (35.32%)	0.742
KPGA	75 (28.41%)	98 (37.12%)	91 (34.47%)	
Age				
Mean (SD)	64.9 (8.2)	64.1 (7.1)	64.3 (7.2)	0.503
< 65	91 (29.07%)	113 (36.1%)	109 (34.82%)	0.290
65–74	74 (27.41%)	99 (36.67%)	97 (35.93%)	
> 75	27 (40.91%)	18 (27.27%)	21 (31.82%)	
Gender of Patient				
Male	35 (30.97%)	37 (32.74%)	41 (36.28%)	0.804
Female	157 (29.29%)	193 (36.01%)	186 (34.7%)	
Race				
White	171 (30%)	202 (35.44%)	197 (34.56%)	0.777
Non-white	21 (26.58%)	28 (35.44%)	30 (37.97%)	
Education				
High school or less	28 (31.82%)	30 (34.09%)	30 (34.09%)	0.907
Some college	53 (26.9%)	70 (35.53%)	74 (37.56%)	
Completed college	47 (29.94%)	60 (38.22%)	50 (31.85%)	
Graduate school	63 (30.88%)	69 (33.82%)	72 (35.29%)	
Patient health personal characteristics				
Health Literacy				
Low	13 (24.07%)	19 (35.19%)	22 (40.74%)	0.560
High	178 (30.07%)	210 (35.47%)	204 (34.46%)	
Subjective Numeracy				
Low	51 (31.88%)	60 (37.5%)	49 (30.63%)	0.707
Medium	61 (28.64%)	77 (36.15%)	75 (35.21%)	
High	79 (28.94%)	92 (33.7%)	102 (37.36%)	
Patient Activation Score				
Level 1 (0–42.5)	24 (48%)	16 (32%)	10 (20%)	0.031
Level 2 (47.4–52.9)	24 (24%)	36 (36%)	40 (40%)	
Level 3 (56.4–66.0)	92 (30.46%)	99 (32.78%)	111 (36.75%)	
Level 4 (68.5–100)	51 (26.29%)	78 (40.21%)	65 (33.51%)	
Krantz Information Scale				
Low	48 (29.27%)	50 (30.49%)	66 (40.24%)	0.385
Medium	67 (28.39%)	87 (36.86%)	82 (34.75%)	
High	77 (31.3%)	92 (37.4%)	77 (31.3%)	
Krantz Behavioral Scale				
Low	51 (27.27%)	63 (33.69%)	73 (39.04%)	0.652
Medium	61 (31.61%)	69 (35.75%)	63 (32.64%)	
High	78 (29.89%)	97 (37.16%)	86 (32.95%)	
Participant health characteristics				
History of Osteoporosis				
Normal	94 (29.38%)	107 (33.44%)	119 (37.19%)	0.713
Osteopenia	63 (30.58%)	74 (35.92%)	69 (33.5%)	
Osteoporosis	35 (28.46%)	49 (39.84%)	39 (31.71%)	
Self-rated Health				
Poor	5 (29.41%)	5 (29.41%)	7 (41.18%)	0.488
Fair	26 (38.24%)	20 (29.41%)	22 (32.35%)	
Good	64 (29.77%)	84 (39.07%)	67 (31.16%)	
Very good	73 (29.8%)	84 (34.29%)	88 (35.92%)	
Excellent	24 (23.08%)	37 (35.58%)	43 (41.35%)	
Number of Chronic Conditions				
None	86 (28.2%)	112 (36.72%)	107 (35.08%)	0.573
1–2	93 (29.71%)	108 (34.5%)	112 (35.78%)	
> 2	13 (41.94%)	10 (32.26%)	8 (25.81%)	
Provider characteristics				
Intervention Assignment				
Intervention	88 (27.85%)	121 (38.29%)	107 (33.86%)	0.323
Usual care	104 (31.23%)	109 (32.73%)	120 (36.04%)	
Patient Provider’s Gender				
Male	68 (32.69%)	68 (32.69%)	72 (34.62%)	0.435
Female	124 (28.12%)	162 (36.73%)	155 (35.15%)	
Gender of patient and provider				
Female providers and female patients	114 (28.64%)	145 (36.43%)	139 (34.92%)	0.799
Female providers and male patients	10 (23.26%)	17 (39.53%)	16 (37.21%)	
Male providers and male patients	25 (35.71%)	20 (28.57%)	25 (35.71%)	
Male providers and female patients	43 (31.16%)	48 (34.78%)	47 (34.06%)	

### Adjusted associations with web portal use

The multivariable logistic regression analyses (Table 3) revealed that overall, web portal use was more likely for patients who were at UI (REF=KP, AOR = 1/0.39 = 2.56, *p*-value< 0.001), younger than 75 (REF = 65–75, AOR = 1/0.67 = 1.49, *p*-value = 0.011), more educated (REF=High school graduate or less, AOR = 1.56 to 1.81, all *p*-values < 0.002), had higher health literacy (AOR = 1.50, *p*-value = 0.014), self-reported osteopenia (REF=Normal, AOR = 1.45, *p*-value = 0.001), and were of the same gender at their provider (AOR = 1.41, *p* value =0.006). Within sites, the associations were again comparable, although *p* values were less significant due to the reduced sample sizes. The C-statistic (AUC) for our logistic regression model is 0.704, which indicates that it is a good model. Also, the p-value for Hosmer-Lemeshow test is 0.270, suggesting that there is no lack of fit.

**Table 3:** Multivariable logistic regression of patient web portal use

Characteristic	AOR (95% CI)	*P*-value
Demographics		
KP (vs. UI) Site	0.39 (0.31, 0.48)*	< 0.001
Age by category		
< 65	1.02 (0.83, 1.25)	0.867
65–74	1 [Reference]	–
≥ 75	0.67 (0.49, 0.91)*	0.011
Female (vs. male) patient	0.80 (0.62, 1.05)	0.105
Non-White (vs. White) race	0.81 (0.61, 1.08)	0.146
Educational level		
High school graduate or less	1 [Reference]	–
Attended college	1.56 (1.18, 2.08)*	0.002
Completed college	1.55 (1.14, 2.11)*	0.005
Attended graduate school	1.81 (1.32, 2.47)*	< 0.001
Patient health mindset characteristics		
Higher health literacy	1.5 (1.09, 2.07)*	0.014
Subjective numeracy		
Higher numeracy	1.19 (0.96, 1.49)	0.114
Medium numeracy	1 [Reference]	–
Lower numeracy	0.91 (0.71, 1.16)	0.442
Patient Activation Score		
Level 1 (0–42.5)	1 [Reference]	–
Level 2 (47.4–52.9)	1.17 (0.79, 1.73)	0.428
Level 3 (56.4–66.0)	1.36 (0.96, 1.93)	0.085
Level 4 (68.5–100)	1.45 (1.00, 2.11)	0.053
Information-seeking in health care		
Higher level	0.89 (0.72, 1.11)	0.316
Medium level	1 [Reference]	–
Lower level	0.79 (0.63, 1)	0.054
Behavioral involvement in health care		
Higher level	1.23 (0.98, 1.53)	0.074
Medium level	1 [Reference]	–
Lower level	0.88 (0.70, 1.11)	0.275
Health characteristics		
History of Osteoporosis		
Normal	1 [Reference]	–
Osteopenia	1.45 (1.16, 1.81)*	0.001
Osteoporosis	1.12 (0.87, 1.44)	0.388
Self-rated Health		
Poor	0.72 (0.39, 1.31)	0.278
Fair	0.94 (0.68, 1.29)	0.703
Good	1 [Reference]	–
Very good	0.94 (0.76, 1.18)	0.607
Excellent	1.08 (0.80, 1.45)	0.633
Number of Chronic Conditions		
None	1 [Reference]	–
1–2	0.92 (0.76, 1.12)	0.426
> 2	0.70 (0.45, 1.08)	0.108
Provider characteristics		
Intervention patient	0.84 (0.70, 1.00)	0.054
Female Provider	0.92 (0.72, 1.17)	0.486
Same Gender of Patient and Provider	1.41 (1.10, 1.80)*	0.006

*indicates significance of *p*=0.05 or less

## Discussion

We used data from a large multi-site, pragmatic randomized controlled trial to examine which patients learned of their DXA results using available patient web portals. Our analysis revealed that most participants (80.9%) never used the patient web portals to view their DXA results. We also found that portal use was more common among patients who were at UI, younger than 75, more educated, had higher health literacy, self-reported osteopenia, and had a provider of the same gender.

These findings are comparable to reports from other studies that have found web portal use rates ranging from 16 to 35% [22–28]. Direct comparisons are difficult, however, because of the varying definitions of web portal use including whether patients were only registered [22, 26, 28], or if they logged in, or if they activated their account [23–25, 27]. Our study was restricted to patients who reported knowing their DXA results and said that they viewed their DXA results using the web portal. We were not, however, able to know if those who did not use the web portal to view their DXA results used the portal for other purposes.

Low rates of web portal use may be due to patients preferring other forms of communication with their providers for getting their test results. While web portals are strongly endorsed [1], patients may not like them [29]. A study of patient preferences for DXA result notification found that 18% of the patients considered it unacceptable to provide either normal or abnormal DXA results over a secure web portal [29]. Perhaps when patients become more familiar with web portals, their acceptance of web portal notification of test results will increase. Learning more about patient’s perceptions of web portals may also lead to increased use, especially among older adults.

We found that after adjusting for other factors, patients > 75 years old had 67% lower odds of using the web portal to view their DXA results. This is consistent with most prior studies of web portal use [22], although Krist et al. reported an anomalous finding that patients 60–69 years old were more likely to use the web portal than patients 18–59 or 70 years old or older [24]. It has been suggested that older patients actually may be more engaged to use a web portal if they have more chronic diseases, office visits, and diagnostic tests to be informed about [24, 30, 31]. For example, Ancker et al. did find that those with chronic conditions were more likely to register for a patient web portal [22]. While we did not find a significant difference in web portal use based on the number of patient comorbidities, we did find that those who self-reported osteopenia had 45% higher odds of viewing their DXA via their web portal results than those with normal bone density. Patients with a history of osteopenia may be more anxious and thus more motivated to get their DXA results.

Unlike other studies [22–24, 26–28], we did not find that race or sex differed between web portal users and non-users. This may be due to the low number of non-Whites, especially at UI, and to having men included in our sample given that osteoporosis is general associated with White females. One of our most interesting findings was that patients with the same gender as their provider had 41% greater odds of using the web portal than those with a provider of a different gender. To our knowledge, only one other study has examined clinician characteristics related to patient web portal use, and they also found that patients with female clinicians were 37% more likely to use a web portal [24]. Others have shown that women are more likely to be earlier adopters of web portals than men [22–24], and clinician adoption of web portals may stimulate patient use [24].

Consistent with other studies [12, 26, 32], we found that more educated patients had between 56 and 81% greater odds and those with higher health literacy had 50% greater odds of using the web portal to view their DXA results than those with lower education and health literacy. We expected this finding because activating, accessing, and navigating a web portal requires patients to read health-related terms. If web portals are to be emphasized and encouraged going forward, developers should keep in mind health literacy when designing web portal interfaces and how test results are communicated to patients.

Finally, there are factors that limit the generalizability of our findings. First, we included 50–64 year olds even though DXA is generally performed on older adults. In a sensitivity analysis restricted to patients > 65 years old (data not shown), however, the associations reported here were comparable although statistical significance may not have been achieved given the reduced statistical power. Second, we relied on patient-reports of how they learned about their DXA results. Due to IRB constraints and time limitations of the two sites’ information technology staff, we were unable to confirm the validity of those patient reports. Third, those who received the intervention DXA result letter may not have accessed their information via a portal because they already received their results. However, we waited four weeks after the participant had their DXA before mailing the result letter to see if they would to allow time for them to attempt to get their results in another manner. Lastly, because we could not determine the number of participants who had activated web portal accounts, the rate of those who did access their DXA results (13.9%) may have been higher if we looked at only those with activated accounts. However, those who did not have an activated account are still considered non-users of web portals and as such did not view their DXA results on the web portal.

Patient web portals offer an efficient and quick vehicle through which clinicians can communicate with patients, and for patients to be more actively involved in their health care. Determining which patients use web portals to view their DXA results is an important step in encouraging web portal use for health communication. We found that older patients and those with less education or lower health literacy or lower levels of patient activation were less likely to use the web portal. Future research should examine the barriers and facilitators for patients accessing web portals and determine their preferences for receiving their DXA results.

## Data Availability

The datasets generated and/or analysed during the current study are not publicly available as we are awaiting publication of major manuscripts but are available from the corresponding author on reasonable request. Data will be made publicly available in 2018. Please email the corresponding author for information regarding the data repository at stephanie-edmonds@uiowa.edu.

## Abbreviations

CI: Confidence Interval
CMS: Centers for Medicare and Medicaid Services
DXA: Dual-energy X-ray absorptiometry
EHR: Electronic Health Records
HIT: Health Information Technology
KPGA: Kaiser Permanente of Georgia
PAADRN: Patient Activation After DXA Result Notification
PAM: Patient Activation Measure
RA: Research Assistant
UAB: University of Alabama at Birmingham
UI: University of Iowa
US: United States
